# Identification of miRNAs Responsive to a Defined Period of Iron Deficiency and Resupply in *Arabidopsis thaliana*

**DOI:** 10.3390/plants15020227

**Published:** 2026-01-11

**Authors:** Qianmiao Zhao, Fei Liu, Jin Xu, Ping Zhang

**Affiliations:** 1 Shanxi Key Laboratory of Germplasm Resources Innovation and Utilization of Vegetable and Flower, College of Horticulture, Shanxi Agricultural University, Taigu 030801, China; z2242852004@163.com; 2 College of Horticulture, Hebei Agricultural University, Baoding 071001, China; liufei_wyx@126.com

**Keywords:** Fe deficiency, Fe resupply, miRNA-mRNA regulatory network, cis-element IDE1

## Abstract

Iron (Fe), as one of the essential micronutrients for plants, plays a pivotal role in regulating growth and development through homeostatic balance. Fe deficiency is a common agricultural stress that causes visible leaf chlorosis and impairs plant growth. In this study, *Arabidopsis thaliana* seedlings grown under Fe deficiency for 4 days were subjected to 6 h Fe resupply via foliar spray or root supply, followed by measurements of chlorophyll fluorescence and metal ion contents in leaves and roots. Fe deficiency significantly reduced Fe levels and the maximum quantum yield of fluorescence (Fv/Fm), while increasing copper (Cu) accumulation in roots. Zinc (Zn) and manganese (Mn) levels were also altered, depending on tissue type. Fe resupply restored Fv/Fm, increased Mn levels, and rebalanced micronutrient content. MicroRNA (miRNA) mediates adaptation to Fe deficiency via post-transcriptional regulation in plants. However, the involved regulatory networks of miRNAs under stress conditions during Fe resupply following deficiency remain poorly understood. These physiological changes prompted us to explore the underlying regulatory networks using miRNA-seq and mRNA-seq. The bioinformatics analysis identified differentially expressed miRNAs responsive to Fe stress, with the Fe-deficiency-specific cis-element IDE1 characterized in their promoter regions. By integrating miRNA-seq and mRNA-seq datasets, we constructed a regulatory network and identified 13 miRNAs harboring IDE1 motifs alongside their functional target genes. Three critical Fe homeostasis modules were proposed—miR396b-LSU2, miR401-HEMA1, and miR169b-NF-YA2—that link Fe homeostasis to chlorophyll synthesis, sulfur (S) responses, and developmental signaling. This study integrates physiological phenotyping with transcriptomic insights to provide a comprehensive view of Fe deficiency and recovery in *Arabidopsis*.

## 1. Introduction

Iron (Fe), an essential micronutrient for all living organisms, plays an indispensable role in fundamental physiological processes during plant growth and development [1]. As a catalytic cofactor of many functional proteins, Fe is essential for mitochondrial respiration, photosynthetic electron transport, ROS scavenging, the tricarboxylic acid (TCA) cycle, and diverse metabolic pathways [1,2]. Fe mainly exists as insoluble Fe (III) precipitates and is abundant in soil. However, Fe shows extremely low solubility under aerobic conditions, which seriously limits the bioavailability of plants [3]. Conversely, anaerobic acidic soils promote excessive Fe accumulation, leading to plant toxicity [4]. Consequently, plant Fe homeostasis is strictly regulated through complex mechanisms including absorption, transport, and circulation [5].

Vascular plants deploy distinct strategies for Fe acquisition. In *Arabidopsis* roots, Ferric reduction oxidase 2 (FRO2) reduces Fe (III) to soluble Fe (II), which is subsequently imported via the high-affinity iron-regulated transporter 1 (IRT1) (strategy I) [6]. Graminaceous species (e.g., wheat) synthesize and secrete phytosiderophores (PSs) from roots; these compounds highly affinity-chelate Fe (III) to form soluble PS-Fe (III) complexes for membrane transport (strategy II) [3]. Fluctuations in Fe availability in the rhizosphere environment lead to reprogramming of plant transcriptional profiles. As the main regulator, the Fe-deficiency-induced transcription factor/basic helix–loop–helix protein 29 (FIT) is essential in response to Fe deficiency. FIT forms heterodimers with other bHLH family proteins (e.g., bHLH38, bHLH39, bHLH100, and bHLH101) and binds to the promoters of FRO2 and IRT1, directly activating their expression to enhance Fe acquisition [7]. Kobayashi et al. found that IDEs (Fe-deficiency-responsive elements) were involved in the Fe-deficiency response in heterogeneous tobacco plants, specifically overexpressing genes related to the response [8]. IDE1/IDE2 functions synergistically to confer root-specific and Fe-deficiency-inducible expression, suggesting an evolutionarily conserved transcriptional module for Fe stress adaptation [8,9].

Interactions between transcription factors (TFs) and cis-acting elements represent a cornerstone of transcriptional regulatory networks [10]. However, precise spatial–temporal control of gene expression in plants often requires additional regulatory layers. MicroRNAs (miRNAs), endogenous 21-23nt non-coding RNAs ubiquitous in eukaryotic plants, provide critical post-transcriptional regulation. MicroRNA-induced silencing complexes (miRISCs) typically recognize and cleave target mRNAs through base-pair complementarity, thereby post-transcriptionally regulating developmental programs and abiotic stress responses in plants [11,12]. Crucially, miRNAs frequently target TF mRNAs, exerting broad influence over transcriptional regulatory networks. For instance, miRNA-mediated Fe-deficiency tolerance in Citrus sinensis is attributable to the enhanced stress resilience conferred by down-regulated miR172 [13]. The miR396/growth-regulating factor (GRF) module orchestrates root architecture, leaf morphogenesis, floral development, and grain traits [14,15], with the miR396b-*GRF6* sub-module specifically implicated in salt tolerance enhancement in rice [16]. The miR169/nuclear factor Y A (NF-YA) module (encoded by 14 MIR169 loci generating four isoforms in *Arabidopsis*) [17] mediates drought responses in rapeseed and tomato, and salt tolerance in maize [18,19,20]. MiR319 targets Teosinte branched1/cycloidea/pcf (TCP) TFs (e.g., TCP2/3/4/10/24), governing leaf and floral development [21,22,23]. MiR390 fine-tunes lateral root development, leaf polarity, and floral morphology by coordinately regulating ERECTA-family receptor kinases and Auxin response factor (ARF)-mediated signaling in response to drought and salinity [24,25]. MiR160 represses ARF10/16/17, modulating light-dependent hypocotyl elongation with sensitivity to brassinosteroid and gibberellin pathways, as evidenced by brassinazole (BRZ) and paclobutrazol (PAC) inhibition [26].

Visible symptoms such as leaf chlorosis are often the first indicators of Fe deficiency in plants. These symptoms result from reduced chlorophyll synthesis and impaired photosystem II (PSII) function, both of which are Fe-dependent processes [26,27]. In *Arabidopsis*, Fe deficiency also leads to changes in other divalent cations such as manganese (Mn), zinc (Zn), and copper (Cu), due to the altered expression of the metal transporters IRT1 and NRAMP1 [28,29]. While the phenotypic and physiological effects of Fe deficiency are well-documented, the molecular mechanisms that coordinate recovery after Fe resupply remain poorly understood.

In this study, we first observed phenotypic recovery (greening) in Fe-deficient *Arabidopsis* seedlings after Fe resupply. We then quantified changes in chlorophyll fluorescence and metal content to assess physiological recovery. These findings led us to investigate the underlying gene regulatory networks using integrated miRNA-seq and mRNA-seq analyses, focusing on Fe-responsive miRNAs and their target genes. Despite progress in elucidating transcriptional (via IDE1/IDE2-TFs) and post-transcriptional (via miRNAs) regulation of stress responses, the interplay between these layers in controlling Fe homeostasis remains poorly understood. In this study, RNA and miRNA sequencing from roots and leaves of *Arabidopsis* with Fe deficiency and Fe resupply were integrated to identify Fe-deficiency-responsive miRNAs, and (IDE)-miRNA-TF-gene models were proposed for post-transcriptional plant regulation of Fe-related genes.

## 2. Results

### 2.1. Phenotypic and Physiological Responses to Fe Deficiency and Resupply

**Figure 1:**
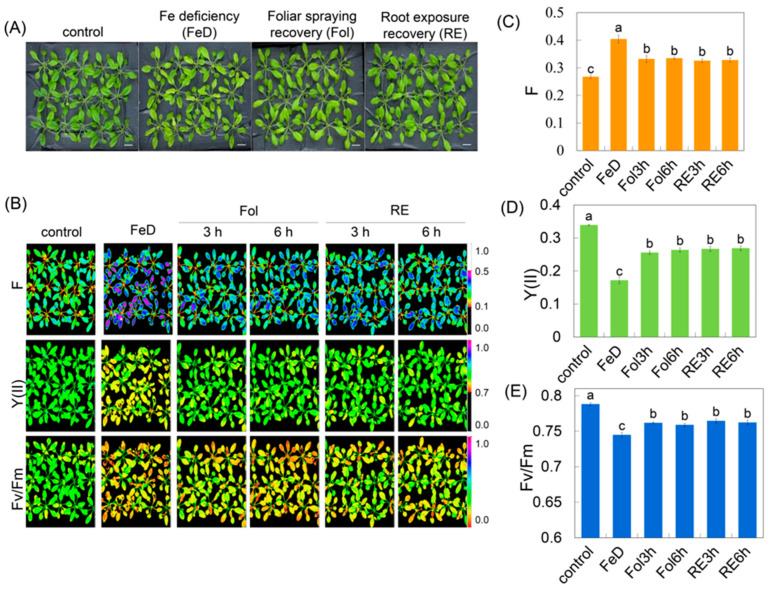
Effects of Fe deficiency and recovery on *Arabidopsis* photosynthetic performance. (**A**) Morphological changes in *Arabidopsis* rosettes under Fe deficiency and recovery treatments. 3-week-old *Arabidopsis*, after 4 d of Fe starvation, was recovered for 6 h by either foliar spraying recovery (Fol) or root exposure recovery (RE). Bar = 1 cm. (**B**) Chlorophyll fluorescence images of *Arabidopsis* leaves under different treatments. Color chlorophyll fluorescence images were captured to visualize the actual fluorescence intensity at any time (F), PSII quantum yield [Y(II)], and maximum quantum yield of fluorescence (Fv/Fm). (**C**–**E**) Quantification of chlorophyll fluorescence parameters under different treatments. F, Y(II), Fv/Fm. The data are presented as mean values with error bars representing the standard deviation. Different letters indicate significant differences among treatments (Duncan’s test, *p* < 0.05).

*Arabidopsis* plants were grown in 1/2 Hoagland solution for a period of 3 weeks and subjected to Fe deficiency for 4 days (d), followed by 6 h of recovery treatments. Two recovery methods were employed: foliar spraying recovery (Fol) and root exposure recovery (RE) (Figure 1A).

Chlorophyll fluorescence imaging revealed a clear Fe-dependent trajectory of PSII performance (Figure 1B). In Fe-sufficient controls, the maximum quantum yield of fluorescence (Fv/Fm) [30] plateaued at 0.78 ± 0.01, while effective PSII quantum yield [Y(II)] and minimum fluorescence (F_0_) remained at basal levels, indicative of a fully oxidized plastoquinone pool and optimal photochemistry (Figure 1B–E). Fe deficiency (FeD) for 4 d elicited a marked drop in Fv/Fm to 0.74 ± 0.01, coinciding with a 40–45% elevation in actual fluorescence intensity at any time (F) and a parallel decline in Y(II) (ΔY = −0.15) (Figure 1C–E), signatures of chronic photoinhibition and diminished photochemical quenching.

**Figure 2:**
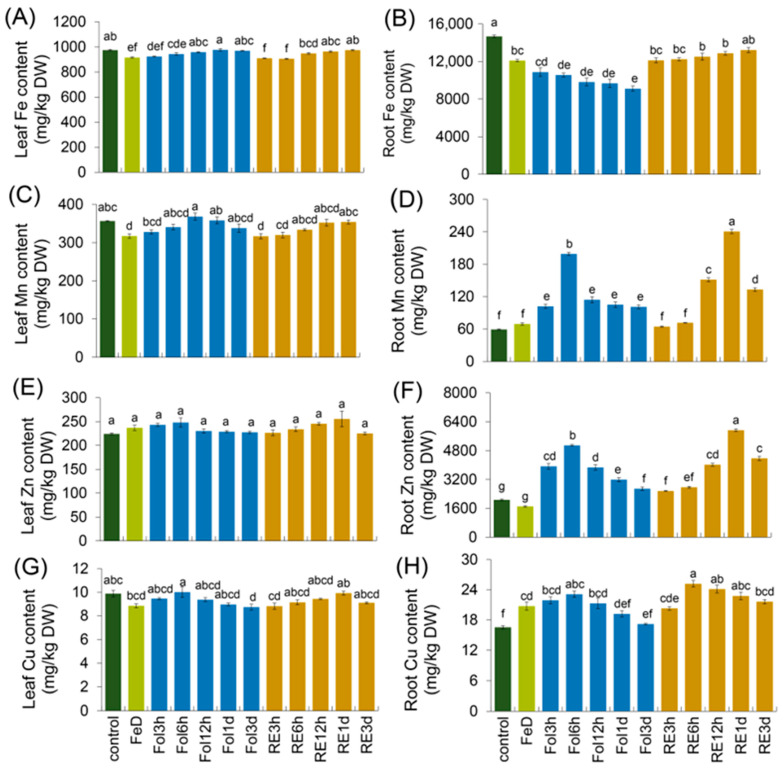
Fe deficiency and recovery alter micronutrient distribution in *Arabidopsis*. *Arabidopsis* plants were grown for 3 weeks in 1/2 Hoagland solution, which was then subjected to Fe deficiency for 4 d, followed by recovery treatments for 3 h, 6 h, 12 h, 1 d, and 3 d using foliar spraying recovery (Fol) and root exposure recovery (RE). (**A**,**C**,**E**,**G**) Fe, Mn, Zn, and Cu contents in leaves under Fe deficiency and recovery treatments. (**B**,**D**,**F**,**H**) Fe, Mn, Zn, and Cu contents in roots under Fe deficiency and recovery treatments. Values are given as the means ±SDs and letters (a, b, c, d, e, f, g) in the figures indicate statistically significant differences among treatments, as determined by Duncan’s test (*p* < 0.05). Groups labeled with the same letter show no significant differences between them.

Fe recovery initiated rapid recovery of the fluorescence parameters (Figure 1B). After 3 h and 6 h of foliar Fe spraying, Fv/Fm climbed to 0.76 ± 0.02, F declined by approximately 19% relative to the Fe-deficiency level, and Y(II) rose proportionally, reflecting the re-opening of PSII reaction centers (Figure 1C–E). Root-exposure Fe recovery produced an equivalent temporal pattern: Fv/Fm reached 0.76 ± 0.02 at 3 h and exhibited no significant change at the 6 h time point, accompanied by a lower F value at both 3 h and 6 h relative to Fe-deficient conditions, and a concomitant incremental gain in Y(II) relative to Fe-deficient conditions (Figure 1C–E). Thus, irrespective of the supply route, Fe resupply rapidly reversed photoinhibition damage, reinstated photochemical quenching, and rebalanced the utilization efficiency of absorbed light energy within PSII. To reveal the mechanistic basis underlying these phenotypic shifts, we quantified the tissue-specific metal ion contents of *Arabidopsis* seedlings exposed to identical Fe treatment conditions. Fe deficiency significantly reduced the Fv/Fm ratio; a lower quantitative value relative to the control indicates pronounced PSII photoinhibition (Figure 1B,E). In addition, we detected the metal ion content under the same treatment; the distribution of Fe and other essential metals showed clear tissue-specific shifts in response to Fe deficiency and subsequent resupply. Fe deficiency resulted in an approximate 19% decrease in Fe content in roots and an 8% decrease in leaves. Following foliar Fe resupply, Fe levels in leaves recovered progressively. By contrast, root Fe content continued to slightly decline from foliar Fe resupply—a pattern that diverged from the gradual recovery observed when Fe was supplied directly to the root (Figure 2A,B). Mn accumulation also differed markedly between roots and leaves. Under Fe deficiency, root Mn content remained similar to the control, while leaf Mn levels decreased compared to the control. This pattern reversed following Fe resupply: root Mn content increased rapidly relative to the control, whereas leaf Mn content recovered to the control levels (Figure 2C,D). Zn and Cu exhibited distinct tissue-specific dynamics under the same experimental conditions. Zn concentrations in leaves and roots were not significantly affected by Fe deficiency. However, Fe resupply triggered a significant increase in Zn accumulation specifically in roots, with no corresponding change in leaves. In contrast, Cu levels rose in roots but declined slightly in leaves under Fe deficiency. After Fe resupply, root Cu content rose transiently before gradually declining, whereas Cu levels in leaves showed little variation throughout the experiment (Figure 2E–H). Upon Fe resupply, Fv/Fm rebounded to values not significantly different from those of the control, and Mn, Zn and Cu concentrations were partially reverted or reduced, with the magnitude of the change potentially depending on the resupply route (root or foliar). These physiological data confirmed that Fe deficiency not only disrupts photosynthetic function but also alters micronutrient balance. The rapid recovery observed after Fe resupply suggests that *Arabidopsis* can quickly rebalance metal homeostasis and restore PSII function, likely through transcriptional and post-transcriptional regulation.

### 2.2. Dynamic Analysis of miRNA Expression Profiles

**Figure 3:**
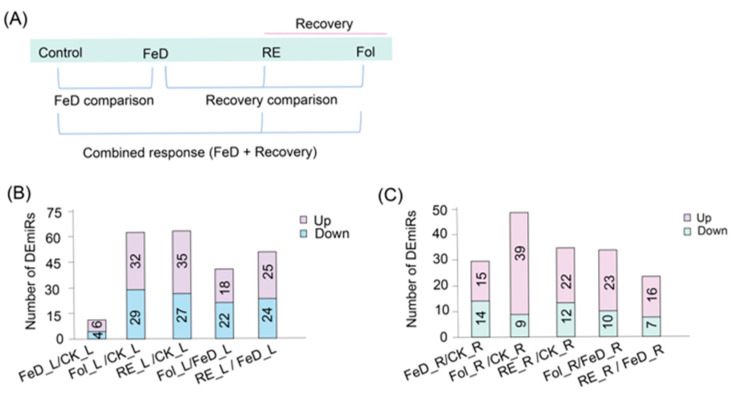
Temporal dynamics of Fe-responsive miRNA expression. (**A**) *Arabidopsis* plants were grown for 3 weeks in 1/2 Hoagland solution, then subjected to 4 d of Fe deficiency, followed by a 6 h recovery treatment using foliar spraying (Fol) and root exposure (RE). The experimental workflow illustrates the root and leaf sampling timepoints under Fe treatments. (**B**,**C**) Differential expression analysis of miRNAs across treatment groups. Significantly dysregulated miRNAs (|log_2_FC| ≥ 0.5 and *p* ≤ 0.05) in leaves (**B**) and roots (**C**) were identified by comparing different treatment groups. These panels show the miRNAs that exhibited significant changes in expression levels in response to Fe deficiency and recovery treatments.

To elucidate the overall changes in miRNA expression profiles in the plant growth process from Fe deficiency to recovery, *Arabidopsis* seedlings were treated with Fe deficiency for 4 d or recovered for 6 h after Fe deficiency, and the roots and leaves were collected for miRNA sequencing (Figure 3A). Differential miRNA profiles define organ-specific Fe responses. In leaves, Fe deficiency (FeD_L vs. CK_L) induced 10 differentially expressed miRNAs (DEmiRs), comprising 6 upregulated and 4 downregulated miRNAs (Figure 3B). Subsequent Fe repletion triggered broader miRNA shifts, and root resupply (RE_L vs. CK_L) elicited 62 DEmiRs (35 upregulated, 27 downregulated), while foliar Fe spray (Fol_L vs. CK_L) induced 61 DEmiRs (32 upregulated, 29 downregulated; Figure 3B). Critically, direct comparison between foliar Fe spray and Fe-deficient leaves shows that root resupply (RE_L vs. FeD_L) triggered 49 DEmiRs (25 upregulated, 24 downregulated), whereas foliar application (Fol_L vs. FeD_L) induced 40 DEmiRs (18 upregulated, 22 downregulated), indicating organ-specific regulatory impacts on miRNA networks (Figure 3B). Parallel analyses in roots revealed Fe deficiency (FeD_R vs. CK_R) induced significant expression changes in 29 miRNAs (15 upregulated, 14 downregulated; Figure 3C). Resupply to deficient roots further modulated miRNA dynamics. Root resupply (RE_R vs. FeD_R) induced 23 DEmiRs (16 upregulated, 7 downregulated; Figure 3C), while foliar application to plants (Fol_R vs. FeD_R) triggered 33 DEmiRs (23 upregulated, 10 downregulated; Figure 3C). Comprehensive comparisons relative to control roots confirmed substantial dynamics, with root resupply (RE_R vs. CK_R) inducing 34 DEmiRs (22 upregulated, 12 downregulated; Figure 3C) and foliar application (Fol_R vs. CK_R) eliciting 48 DEmiRs (39 upregulated, 9 downregulated; Figure 3C). Collectively, these spatially resolved datasets demonstrate pronounced heterogeneity in organ-specific miRNA dynamics under Fe deficiency and resupply interventions.

### 2.3. Identification of Fe-Deficiency-Responsive cis-Regulatory Elements

Building on the paradigm that functionally related genes share cis-regulatory logic, we hypothesized that Fe-responsive miRNAs may harbor conserved Fe-deficiency-responsive elements (IDEs) in their promoters. To test this, promoter sequences of *Arabidopsis* miRNA genes were retrieved from the TAIR database and systematically screened for IDE1 and IDE2 motifs using established homology-based algorithms (BLASTN, 2.15.0.) [31]. The initial validation confirmed the presence of phylogenetically conserved IDE motifs (primarily IDE1) within the promoters of canonical Fe-deficiency induced genes (e.g., *FIT1*, *FRO2*, and *IRT1*) (Supplementary Table S1), aligning with prior reports in graminaceous species [9]. Given the compact architecture of *Arabidopsis* primary miRNA transcripts, core promoters typically lie within ~2 kb upstream of the pre-miRNA hairpin; we focused our analysis on these TSS-proximal regions. Strikingly, 13 miRNA genes contained high-confidence IDE1 homologs (Table 1, Supplementary Figure S1), exhibiting significant sequence conservation. Notably, IDE2 motifs were undetectable across all screened miRNA promoters, indicating selective evolutionary conservation of IDE1-mediated regulation within the Fe-responsive miRNA cohort (Table 1). Of the 13 IDE1-containing miRNAs, Fe stress and resupply drive leaf-specific, root-specific or pan-organ expression, revealing discrete tissue patterns in *Arabidopsis*, miR396b-5p (AT5G35407), miR390a-3p (AT2G38325), miR160c-3p (AT5G46845), miR167c-5p (AT3G04765), and miR319c (AT2G40805) were detected in both roots and leaves; root-specific miRNAs included miR394a (AT2G40805), miR171c-5p (AT1G62035), miR158b (AT1G55591), miR169b-5p(AT5G24825), and miR167a-5p (AT3G22886); leaf-enriched miRNAs comprised miR167a-3p (AT3G22886), miR401 (AT4G08116), and miR396b-3p (AT5G35407).

### 2.4. Transcriptomic Analysis

**Figure 4:**
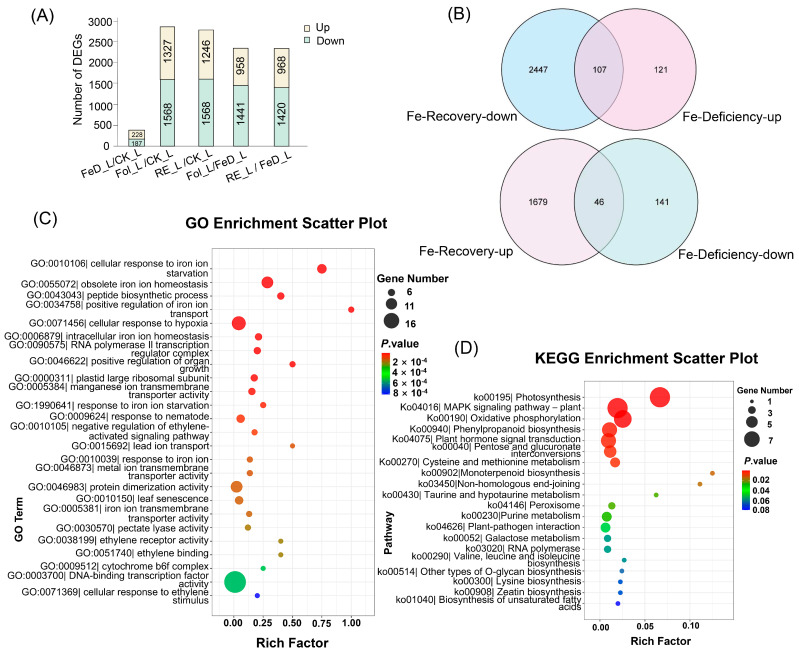
Leaf transcriptome profiling of *Arabidopsis* plants grown for 3 weeks and then subjected to Fe deficiency for 4 d (FeD_L), root exposure recovery for 6 h (RE_L), and foliar spraying recovery for 6 h (Fol_L). (**A**) Count of differentially expressed genes (DEGs) in leaves among the five groups filtered by |log_2_FC| ≥ 1 and *p* ≤ 0.05. (**B**) Venn diagram of DEGs exhibiting opposite expression trends between Fe deficiency and Fe recovery (RE_L and Fol_L); the intersecting genes were defined as Fe-responsive DEGs in leaves. (**C**) Gene Ontology (GO) biological process enrichment analysis of Fe-responsive DEGs in leaves. (**D**) Kyoto Encyclopedia of Genes and Genomes (KEGG) pathway enrichment analysis of Fe-responsive DEGs in leaves.

**Figure 5:**
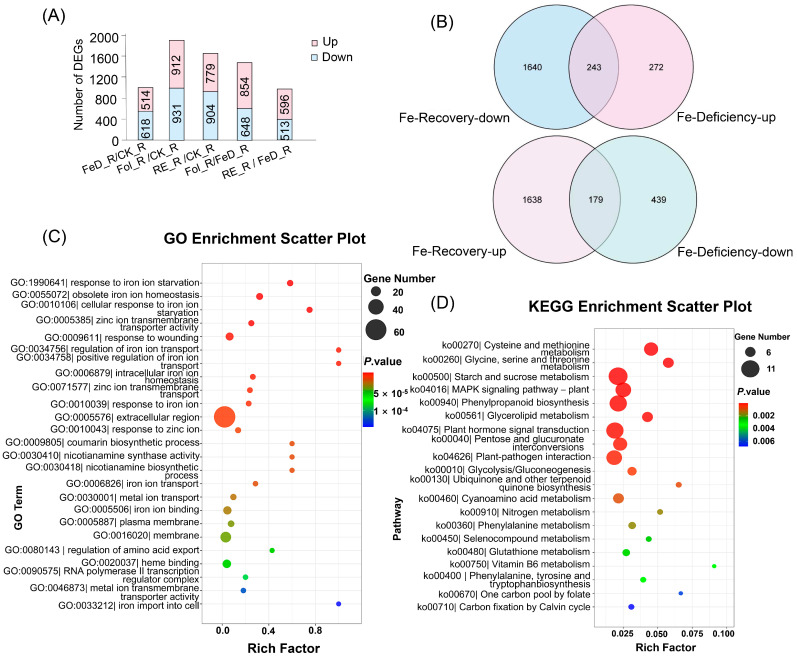
Root transcriptome profiling of *Arabidopsis* plants grown for 3 weeks and then subjected to Fe deficiency for 4 d (FeD_R), root exposure recovery for 6 h (RE_R), and foliar spraying recovery for 6 h (Fol_R). (**A**) Count of differentially expressed genes (DEGs) in roots among the five groups filtered by |log2FC| ≥ 1 and *p* ≤ 0.05. (**B**) Venn diagram of DEGs exhibiting opposite expression trends between Fe deficiency and Fe recovery (RE_R and Fol_R); the intersecting genes were defined as Fe-responsive DEGs in roots. (**C**) Gene Ontology (GO) biological process enrichment analysis of Fe-responsive DEGs in roots. (**D**) Kyoto Encyclopedia of Genes and Genomes (KEGG) pathway enrichment analysis of Fe-responsive DEGs in roots.

RNA-seq of *Arabidopsis* leaves and roots across eight Fe regimes captured genome-wide responses, control (CK_L, CK_R), Fe-deficient (FeD_L, FeD_R), root-resupplied (Re6h_L, Re6h_R), and foliar-resupplied (Fol_L, Fol_R) tissues. Principal component analysis (PCA) revealed robust segregation of samples by treatment. In the root samples, the first principal component (PC1) and the second principal component (PC2) accounted for 45.26% and 26.02% of the variance, with corresponding eigenvalues of 5.4312 and 3.1224, respectively. On the other hand, in leaf samples, PC1 and PC2 explained 74.16% and 8.93% of the variance, with corresponding eigenvalues of 8.8992 and 1.0716, respectively (Supplementary Figure S2), confirming organ-specific *Arabidopsis* Fe responses. Complementary differential expression analysis identified pronounced Fe-dependent dynamics. In leaves, Fe deficiency (FeD_L vs. CK_L) induced 415 differentially expressed genes (DEGs) (228 upregulated/187 downregulated; Figure 2A), while resupply elicited massive transcriptomic shifts, root resupply (RE_L vs. CK_L) generated 2814 DEGs (1246 upregulated/1568 downregulated; Figure 4A) and foliar resupply (Fol_L vs. CK_L) 2895 DEGs (1327 upregulated/1568 downregulated; Figure 4A). Direct comparison to deficiency states further highlighted resupply dynamics: RE_L vs. FeD_L induced 2388 DEGs (968 up/1420 down; Figure 4A) versus Fol_L vs. FeD_L with 2399 DEGs (958 up/1441 down; Figure 4A). Root tissues exhibited parallel but attenuated responses. FeD_R vs. CK_R yielded 1132 DEGs (514 upregulated/618 downregulated; Figure 5A), RE_R vs. CK_R 1683 DEGs (779 upregulated/904 downregulated; Figure 5A), and Fol_R vs. CK_R 1843 DEGs (912 upregulated/931 downregulated; Figure 5A). Resupply-driven changes relative to deficiency were more moderate (RE_R vs. FeD_R: 1109 DEGs [596 upregulated/513 downregulated]; Fol_R vs. FeD_R: 1502 DEGs [854 upregulated/648 downregulated]; Figure 5A).

Filtering identified core Fe-responsive gene sets inversely regulated across conditions: 515 root and 228 leaf transcripts were consistently upregulated during deficiency, but 1883 root and 2554 leaf transcripts were downregulated following supplementation (Figure 4B and Figure 5B). Strikingly, 243 root and 107 leaf genes exhibited this antagonistic expression pattern across both conditions, signifying central roles in Fe homeostasis (Figure 4B and Figure 5B). Similarly, 618 (roots) and 187 (leaves) genes were suppressed during Fe deficiency, but 1817 (roots) and 1725 (leaves) genes were induced upon Fe resupply, with 179 and 46 genes, respectively, displaying this conserved regulatory logic (Figure 4B and Figure 5B).

The functional enrichment of these conserved Fe-responsive genes revealed coordinated regulatory mechanisms. Analysis of the Gene Ontology (GO) biological process highlighted enriched DNA-binding transcription factor activity (GO:0003700) and ethylene receptor activity (GO:0038199) in leaves, while both organs showed significantly enriched responses to Fe ion starvation (GO:0010106) and obsolete Fe ion homeostasis (GO:0055072) (Figure 4C and Figure 5C). Kyoto Encyclopedia of Genes and Genomes (KEGG) pathway analysis further identified iron-dependent co-regulation of plant hormone signal transduction (ko04075), MAPK signaling pathway (ko04016), photosynthesis (ko00195), and starch and sucrose metabolism (ko00500) across tissues (Figure 4D and Figure 5D). These evolutionarily conserved modules demonstrate how Fe perturbations trigger integrated signaling cascades that rebalance metabolism, growth, and stress adaptation through spatiotemporally coordinated transcriptional networks.

### 2.5. Prediction of the Targets of miRNAs Containing IDE1 and Functional and Pathway Analysis of the Target Genes

**Figure 6:**
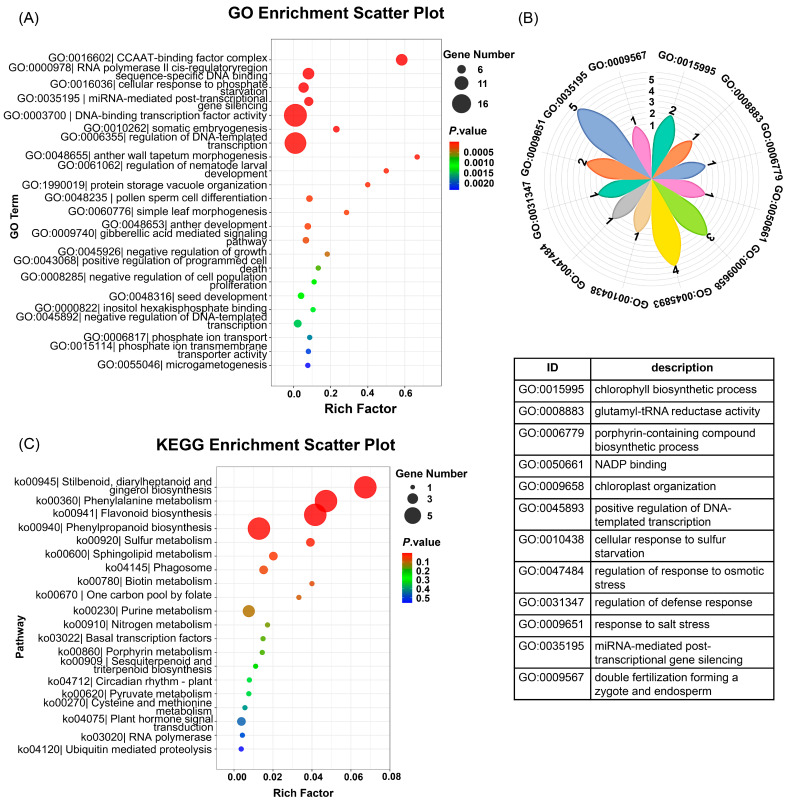
Functional enrichment of target genes predicted for differentially expressed IDE1-containing miRNAs after Fe deficiency for 4 d (FeD_L, FeD_R), root exposure recovery for 6 h (RE_L, RE_R) and foliar spraying recovery for 6 h (Fol_L, Fol_R). (**A**) Gene Ontology (GO) biological process enrichment analysis of the 13 IDE1-containing miRNAs’ target genes; (**B**) magnified petal-diagram view of the GO terms that were partially hidden in panel (**A**), with the table listing the specific GO biological processes. The numbers on the petal diagram represent the number of genes enriched in the specific GO biological processes; (**C**) Kyoto Encyclopedia of Genes and Genomes (KEGG) pathway enrichment of the IDE1-containing miRNAs’ target genes.

Building on the identification of 13 IDE1-harboring miRNAs, we computationally predicted their downstream targets using PsRobot (v1.2) with *Arabidopsis* genome annotations. This stringent analysis identified 118 high-confidence target genes (Supplementary Table S2). Functional enrichment of these targets revealed their profound involvement in transcriptional and post-transcriptional regulation. GO analysis demonstrated significant enrichment for key biological processes: DNA-templated transcription regulation, miRNA-mediated post-transcriptional gene silencing (GO:0035195), and DNA-binding transcription factor activity (GO:0003700). Additionally, targets were implicated in specific macromolecular complexes (CCAAT-binding factor complex, GO:0016602), metabolic pathways (chlorophyll biosynthetic process (GO:0015995) and glutamyl-tRNA reductase activity (GO:0008883)), stress adaptation (cellular response to S starvation, GO:0010438), and developmental programs (double fertilization forming a zygote and endosperm, GO:0009567) (Figure 6A,B). Complementarily, KEGG pathway analysis highlighted central roles in plant hormone signal transduction (ko04075), S metabolism (ko00920), and specialized metabolism (i.e., flavonoid biosynthesis, ko00941) (Figure 6C). This integrated network topology demonstrates that Fe flux reprograms cellular priorities by suppressing growth-associated transcription while activating stress-adaptive machinery and metabolic reconfiguration.

We cross-referenced the 118 predicted targets with our organ-specific Fe-responsive DEGs to functionally link miRNA-target interactions to FE homeostasis. Notably, three miRNAs exhibited significantly altered expressions of their cognate target genes under Fe perturbations (elaborated below). Intriguingly, though the targets of the remaining ten miRNAs showed no statistically significant differential expression, their associations with S metabolism, flavonoid synthesis, and developmental regulators suggest potential roles in fine-tuning Fe stress responses through conserved metabolic or signaling crosstalk. This establishes three experimentally supported regulatory modules alongside a broader network of putatively Fe-sensitive miRNA-target interactions.

**Figure 7:**
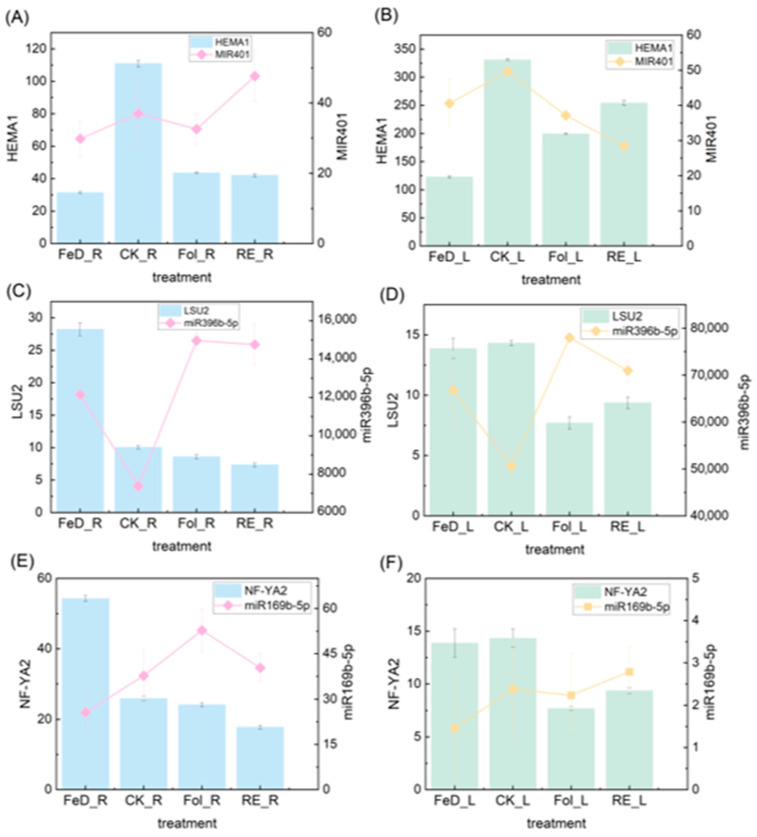
Regulatory-module expression analysis of IDE1-containing miRNAs and their target genes. (**A**–**F**) Left y-axes show the expression of the indicated transcription factor (TF) genes (bars); right y-axes show miRNA expression levels (lines). Expression dynamics of miR401–HEMA1 ((**A**): roots, (**B**): leaves), miR396b–LSU2 ((**C**): roots, (**D**): leaves), and miR169b–NF-YA2 ((**E**): roots, (**F**): leaves) under Fe deficiency for 4 d (FeD_L, FeD_R) followed by root-exposure recovery for 6 h (RE_L, RE_R) and foliar-spraying recovery for 6 h (Fol_L, Fol_R). Values are given as the means ± SDs; significant differences among treatments for both miRNAs and target TFs were determined using Duncan’s test at *p* < 0.05.

Detailed characterization of three prioritized miRNA-target modules revealed sophisticated spatial regulation of Fe responses. The miR401-Glutamyl-tRNA reductase family protein (HEMA1) module (PsRobot score = 2.0, Supplementary Table S3) exhibited antagonistic co-regulation across organs. In leaves, Fe resupply (RE_L/Fol_L vs. FeD_L) downregulated miR401 while upregulating its target HEMA1 (AT1G58290), encoding glutamyl-tRNA reductase—a rate-limiting enzyme in chlorophyll biosynthesis [32]. Although both foliar and root Fe supplementation deliver Fe to the plant, it was observed that miR401 expression was more strongly downregulated and HEMA1 expression was more strongly upregulated during root Fe supplementation (RE_L) compared to foliar Fe supplementation (Fol_L). Interestingly, in roots, compared to Fe deficiency (FeD_R), miR401 expression was significantly upregulated during root Fe supplementation (RE_R) compared to foliar Fe supplementation (Fol_R), while HEMA1 expression showed a downward trend. Therefore, we conclude that the miR401-HEMA1 module exhibits opposite expression patterns in roots and leaves following Fe supplementation. Furthermore, the changes in the expression of this module were more pronounced during root Fe supplementation (RE_L, RE_R) compared to foliar Fe supplementation (Fol_L, Fol_R) (Figure 7A,B). This may be related to Fe transport within the plant and tissue-specific expression differences, but more significantly indicates the fine-tuned regulation of Fe homeostasis in plants.

Parallel analysis of the miR396b response to low sulfur 2 (LSU2) module (PsRobot score = 2.5, Supplementary Table S3) demonstrated conserved miRNA-target repression but divergent organ sensitivity. LSU2 (AT5G24660), a sulfur (S)-starvation-responsive TF [33], was downregulated during Fe resupply concomitant with miR396b upregulation in both organs (Figure 7C,D). However, depending on the site of supplementation, miR396b expression was more downregulated and LSU2 expression was more upregulated during root Fe supplementation (RE_L) compared to foliar Fe supplementation (Fol_L) (Figure 7D). Therefore, the miR396b-LSU2 module still shows distinct expression patterns between roots and leaves. This suggests a potential crosstalk between Fe and S metabolism requiring coordinated regulation across organs.

Complementarily, the miR169b-Nuclear transcription factor y subunit A2 (NF-YA2) module (PsRobot score = 2.5, Supplementary Table S3) showed nuanced organ-specific dynamics. NF-YA2 (AT3G05690) is a CCAAT-box binding TF regulating flowering time and stress responses [34,35]. In roots, compared to Fe supplementation (Fol_R, RE_R), miR169b expression was downregulated, and NF-YA2 expression was upregulated under Fe deficiency (FeD_R). In both roots and leaves, compared to Fe deficiency (FeD_L, FeD_R), miR169b expression was generally upregulated during both foliar Fe supplementation (Fol_L, Fol_R) and root Fe supplementation (RE_L, RE_R), while NF-YA2 expression was generally downregulated. Nevertheless, differences were observed based on the supplementation organ; miR169b expression was higher in RE_L compared to Fol_L but lower in RE_R compared to Fol_R (Figure 7E,F). Therefore, we conclude that while the overall expression pattern of the miR169b-NF-YA2 module appears similar in roots and leaves (upregulation of miRNA and downregulation of target upon Fe supplementation), miR169b expression still exhibits differences depending on the organ receiving the Fe supplement. These modules underscore the spatial and temporal complexity of miRNA-mediated Fe metabolism regulation in *Arabidopsis*.

Collectively, these modules exemplify how plants deploy spatially partitioned regulatory circuits: miR401-HEMA1 coordinates metabolic transitions across source-sink organs, miR396b-LSU2 integrates Fe-S nutrient sensing, and miR169b-NF-YA2 balances stress adaptation with developmental timing all fine-tuned by Fe translocation pathways.

## 3. Discussion

Fe deficiency induces yellowing of *Arabidopsis* leaves [7]. After 4 d of Fe deficiency, we observed only faint chlorosis. We propose that chlorosis intensifies with prolonged Fe deprivation and that leaves re-green rapidly upon Fe resupply. This rapid phenotypic recovery was mirrored by measurable changes in PSII efficiency and metal homeostasis.

Previous studies have established that Fe deficiency up-regulates the expression of IRT1 and NRAMP1, which in turn mediate the uptake of Zn^2+^, Mn^2+^ and other metal ions [28,29]. Nevertheless, our results did not detect any significant changes in Zn^2+^ or Mn^2+^ under Fe-deficient conditions in roots. Castaings et al. (2016) have shown that IRT1 and NRAMP1 persist under Fe-sufficient conditions and continue to mediate metal-ion transport [36]. In our experiments, resupplying Fe increased root Zn^2^ and Mn^2+^ contents. During this interval, we suggest that the still-present IRT1 and NRAMP1 continued to transport extracellular Zn^2+^ and Mn^2+^ into the root, producing the observed peak. Previous studies observed a root/leaf Fe ratio of ~10:1 in hydroponic *Arabidopsis* supplied with 25–40 μM Fe, identical to that in our dataset [36,37]. Castaings et al. (2016) showed that shoot Fe rises linearly from 40 to 100 µg g^−1^ at 25 µM to ~300 µg g^−1^ at 500 µM or 1 mM [36]. In this study, at 50 μM Fe, Fe contents exceed those reported at 25 μM, consistent with the linear increase documented by Castaings et al. (2016) [36]. Under the same 50 μM Fe supply, Barberon et al. (2014) reported root Fe contents of 200 μg mg^−1^ under Fe deficiency and 800 μg mg^−1^ under Fe sufficiency on MS agar plates, while Wang et al. (2013) observed 80 mg g^−1^ under Fe deficiency and 200 mg g^−1^ under Fe sufficiency on MS agar plates [38,39]. Although the absolute Fe levels we report differ from those in the literature, such discrepancies can be ascribed to variations in treatment conditions.

The drop in Fv/Fm confirmed that Fe deficiency directly impairs photosynthetic performance, consistent with previous reports [40]. The fact that these physiological changes were reversed within 6 h of Fe resupply suggests that *Arabidopsis* possesses a rapid, finely tuned regulatory system to sense and respond to Fe availability. To uncover the molecular basis underlying these physiological responses, we performed integrated miRNA-seq and mRNA-seq. Three-week-old seedlings were first subjected to 4 d of Fe deficiency, then sprayed with 25 µM Fe-EDTA or transferred to fresh 1/2 Hoagland solution containing 50 µM Fe-EDTA and harvested 6 h later. This brief period captures the maximum early transcriptional response while avoiding secondary effects [41,42]. By integrating miRNA-seq and mRNA-seq, we identified key miRNA–mRNA modules that not only respond to Fe status but also regulate genes involved in chlorophyll synthesis (HEMA1), sulfur-responsive (LSU2), and developmental signaling (NF-YA2). Thus, our omics data provide a mechanistic explanation for the observed physiological recovery, linking Fe homeostasis to broader stress adaptation networks.

Plant Fe homeostasis represents a critical frontier where miRNA-target modules function as synergistic regulators of nutrient signaling. While miRNAs are established post-transcriptional regulators of mineral stress responses [43], their specific roles in Fe metabolism remain underexplored. IDE1 and IDE2, as Fe-deficiency-responsive cis-regulatory elements, are widely recognized as the canonical motifs governing plant responses to Fe limitation [8,9]. Therefore, from 13 Fe-responsive miRNAs through IDE1 cis-element screening in *Arabidopsis* roots and leaves, we systematically identified differential miRNAs [31,44]. Integrated miRNA-Seq/RNA-Seq analyses demonstrated inverse expression patterns between candidate miRNAs and their targets under Fe resupply, potentially suggesting functional miRNA-target modules in Fe homeostasis.

Three core regulatory modules emerged: (1) In tetrapyrrole biosynthesis, Fe deficiency downregulated miR401 in leaves to upregulate HEMA1, encoding glutamyl-tRNA reductase, the NADPH-dependent rate-limiting enzyme in chlorophyll biosynthesis [32,45]. Given the limited research on miR401, we hypothesize that it upregulates chlorophyll synthesis to coordinate Fe allocation toward essential pathways like respiration during Fe deficiency. (2) In Fe-S metabolic crosstalk, Fe resupply induced miR396b in roots while repressing LSU2, an S-starvation transcription factor that reprograms S metabolism during cadmium (Cd) stress [33,46]. This extends beyond miR396’s canonical growth regulation via GRF targeting [14,15,16], suggesting the role of LSU2 in maintaining functionality under Fe fluctuations. (3) In developmental regulation, miR169b was downregulated under Fe deficiency and upregulated NF-YA2, a subunit of the heterotrimeric NF-Y complex regulating stress responses and root architecture [19,35]. This aligns with miR169/NF-YA’s documented roles in drought/salt stress across species [17,18,19,20,47,48], exemplifying the integration of Fe signaling with broader stress networks. Collectively, these modules demonstrate miRNA-mediated prioritization of Fe allocation (miR401-HEMA1), coordination of Fe-S metabolic flux (miR396b-LSU2), and recalibration of development-stress tradeoffs (miR169b-NF-YA2) under fluctuating Fe availability conditions. This framework positions miRNAs as central orchestrators of Fe homeostasis through targeted repression of rate-limiting enzymes, metabolic regulators, and developmental transcription factors. Additionally, the remaining 10 miRNAs were subjected to analysis. Our identification of IDE1-containing miRNAs extends their established regulatory networks through novel target interactions. Specifically, miR158b targets the pentatricopeptide (PPR) repeat, corroborating the conserved role of miR158 in suppressing PPR transcripts since its initial discovery [49] and in its regulation of pollen development [50]. Similarly, miR167c targets ARF6 and ARF8, reinforcing miR167’s well-characterized repression of auxin signaling components [51,52] to govern growth-defense trade-offs. Novel metabolic regulation is evidenced by miR171c targeting *APR3*, an *S*-assimilation rate-limiting enzyme [53], which, combined with its known repression of SCL6-mediated shoot branching [54,55], suggests coordinated control of S metabolism and development under Fe deficiency.

Further functional expansion is observed in miR319c, which targets MYB33 and MYB65, extending beyond canonical TCP transcription factor regulation (TCP2/3/4/10/24) in morphogenesis [21,22,23]. This functional parallel with miR159-mediated MYB suppression [56] is suggestive of the combinatorial miRNA control of stress adaptation. Receptor-based regulation is highlighted by miR390a targeting ERL1 (AT5G62230), which operates alongside the canonical TAS3-ARF2/3/4 pathway [25] to coordinate stomatal development, stress resilience (thermotolerance/drought/pathogen defense), and auxin-mediated organogenesis.

Our study identifies a Tetratricopeptide repeat (TPR)-like superfamily protein as a novel target of miR394a, given the established role of TPR proteins in coordinating abiotic stress responses across plant species—including drought and ABA signaling, as demonstrated in tomatoes [57]. We propose that the parallel dual-targeting mechanisms, which include miR394a suppressing a TPR-like abiotic stress sensor while co-regulating LCR-mediated leaf morphogenesis [58], potentially mitigate Fe stress through developmental plasticity. Conservation is further evidenced by miR167a targeting ARF8, validating cross-species miR167-ARF6/8 regulation [51,59] in maintaining auxin homeostasis under Fe stress. Notably, our study reveals ADR1-like 1 as a novel target of miR160c-3p, which targets the immune regulator ADR1-like 1 [60,61], converging with its canonical repression of ARF10/16/17-mediated hypocotyl elongation to position this miRNA as a signaling hub, rewiring defense and growth programs under environmental fluctuation [26].

It is important to note that the relatively small changes in Fe content observed at the whole-organ level in this study may mask significant tissue- or cell-type-specific redistribution of Fe. Previous studies have shown that Fe can be preferentially accumulated or depleted in specific tissues or cell layers—such as the vasculature or root apex—even when total Fe content in the organ remains unchanged [62,63]. Integrating prior molecular evidence with our new data, we propose a miRNA–TF co-regulatory network for Fe metabolism (Supplementary Figure S3). This model clarifies nutrient-homeostasis control and guides molecular breeding toward Fe-biofortified crops.

## 4. Materials and Methods

### 4.1. Plant Material and Experiment Treatments

The seeds of wild-type *Arabidopsis thaliana* (Col-0) were sterilized with 50% (*v*/*v*) bleach. After surface sterilization, the seeds were rinsed with sterile water in a laminar flow hood five times and spotted on 1/4 MS basic medium (pH = 5.75) containing 1% (*w*/*v*) sucrose and 0.8% (*w*/*v*) agar. The seeds were stratified at 4 °C for 2 d; plates were then incubated vertically in a phytotron. The culture conditions throughout the experiment were as follows: all plants were grown under 120 μmol m^−2^ s^−1^ Philips Green Power LEDs (Amsterdam, The Netherlands), with a 16 h light/8 h dark cycle at 22 °C (light) and 19 °C (dark). Five-day-old *Arabidopsis* seedlings were transferred to 1/2 Hoagland solution (5 mM Ca(NO_3_)_2_, 2 mM MgSO_4_, 5 mM KNO_3_, 1 mM NH_4_H_2_PO_4_, 50 μM Fe(III)-EDTA, 3 μM H_3_BO_3_, 1 μM (NH_4_)_6_MO_7_O_24_, 0.4 μM ZnSO_4_, and 0.2 μM CuSO_4_) (replaced every 5 d) and grew for 3 weeks. The prepared seedlings were transferred to 1/2 Hoagland solution with or without Fe-EDTA for 4 d, and then transferred to fresh 1/2 Hoagland solution or foliar spraying with 25 μM Fe(III)-EDTA solution for 6 h, as a control for the foliar Fe spraying. Seedlings that had likewise been grown under Fe deficiency for 4 d were foliar-sprayed with the same volume of distilled water.

### 4.2. miRNA and RNA Sequencing and Bioinformatics Analysis

Total RNA was extracted using TRlzol Reagent (Takara, Beijing, China). The miRNA library was prepared according to Illumina’s instructions and sequenced on the Illumina Hiseq2500 platform (Illumina, San Diego, CA, USA). Based on the *Arabidopsis* data in miRBase 22.0 [64], ACGT101-miR (LC Houston, Houston, TX, USA) was used to filter the raw sequencing reads. The differential expression of miRNA was analyzed based on normalized deep-sequencing counts [65]. To accommodate sparse read coverage, we relaxed the differential expression threshold for miRNAs to |log_2_FC| ≥ 0.5 (from the default |log_2_FC| ≥ 1) while retaining statistical significance (*p* ≤ 0.05) [66]. The PsRobot (v1.2) algorithm was used to predict the potential target genes of DEmiRs (score < 2.5) [67,68]. For RNA sequencing, differential expression analysis was performed using DESeq2 (adjusted *p* ≤ 0.05, log_2_FC ≥ 1 or log_2_FC ≤ −1) [69]. Based on the GO database (http://www.geneontology.org/ (accessed on 10 July 2025)) and KEGG database (http://www.genome.jp/kegg/ (accessed on 10 July 2025)), we performed functional annotation and pathway analysis on potential DEGs, respectively. The miRNA-seq and mRNA-seq data were submitted to NCBI (SRA accession numbers: SUB12231786 and SUB15654147). Principal component analysis (PCA) was performed with the built-in R function prcomp (stats package v3.5.0; scale = TRUE). Group separation was statistically evaluated using anosim from the vegan package (v2.5.4). Confidence ellipses were drawn with stat_ellipse in ggplot2 v3.2.0.

### 4.3. Identification of IDE in DemiRs

Genome sequences were retrieved from the TAIR database (https://www.Arabidopsis.org/ (accessed on 10 July 2025)). BLASTN was applied to identify homologous sequences of IDE1 and IDE2. For IDE1, composed of two conserved 9 bp modules, stringent homology thresholds were applied, with ≥6/9 bp (≥66.7%) perfect matches in each module. For IDE2, which contains three modules, the central 9 bp core was required to exhibit ≥6/9 bp (≥66.7%) conservation, while flanking regions allowed ≥14/27 bp (51.8%) overall conservation. Specifically, flanking 9 bp modules required ≥6/9 bp (≥66.7%) homology, with nucleotide variations permitted in regions beyond 6 bp from the core [31]. The hierarchical criteria balanced the structural integrity of functional domains with evolutionary sequence polymorphism.

### 4.4. Phenotypic Observation and Imaging

*Arabidopsis* seedlings were photographed under standardized light conditions using a Canon EOS 90D (60 mm macro lens, 5600 K light (Canon inc., Ōita, Japan)) at 25 cm. Chlorosis was quantified in ImageJ (v1.53) by extracting the mean hue angle (H°) from the central leaflet (≥5000 pixels); H° > 10° shift into 55–70° was scored as significant chlorosis [70]. A color checker (X-Rite Mini) was used for white-balance correction in each frame.

### 4.5. Chlorophyll Fluorescence Parameters

Chlorophyll fluorescence parameters were recorded with an Imaging-PAM imaging fluorometer (Heinz Walz GmbH, Effeltrich, Germany). Three-week-old *Arabidopsis* seedlings were transferred to fresh 1/2 Hoagland solution for either normal nutrient supply (control) or Fe deficiency for 4 d, then transferred to fresh 1/2 Hoagland solution or foliar-sprayed with 25 μM Fe-EDTA solution for 6 h. Prior to measurement, following 30 min dark adaptation at 25 °C, a saturation pulse (4000 µmol photons m^−2^ s^−1^, 0.8 s) was applied to obtain minimum fluorescence (F_0_) and maximum fluorescence (Fm). The fluorescence parameters were calculated using a previously reported method [71] and the equations provided therein. The maximum quantum efficiency of PSII was calculated as Fv/Fm = (Fm − F_0_)/Fm. Thereafter, actinic light (120 µmol·m^−2^·s^−1^) was switched on, and images were captured after 5 min steady-state photosynthesis to determine the effective quantum yield of PSII [Y(II) = (Fm′ − Fs)/Fm′], where Fm′ is the maximum fluorescence under light and Fs is the steady-state fluorescence. Actual fluorescence intensity at any time (F) was read directly from the first image prior to the saturation pulse.

### 4.6. Determination of Trace Element Contents

*Arabidopsis* seedlings were subjected to 4 d of Fe-deficiency treatment followed by 6 h of root exposure to Fe recovery or foliar Fe spraying. Then, seedlings were soaked in 1 mM Na_2_EDTA for 30 min and rinsed five times with ddH_2_O. The roots and leaves were harvested, dried at 70 °C to constant weight, and ground to a fine powder [72]. The dried samples (0.3 g) were then digested in HNO_3_ (65–68%, (*w*/*w*)), Shanxi Tongjie Chemical Reagent Co., Ltd., Shanxi, China). Samples were completely digested using a microwave digestion system (REVO, LabTech, Beijing, China), and Cu, Zn, Fe, and Mn concentrations were subsequently quantified by inductively coupled plasma mass spectrometry using an iCAP Q ICP-MS (Thermo Fisher Scientific, Waltham, MA, USA).

## Figures and Tables

**Table 1:** *Arabidopsis* miRNAs with promoter IDE1 motifs predicted to regulate Fe-deficiency/recovery responses.

miRNA Gene	IDE Motifs	Location of IDE1-Like	Chromosomal Location
miR158b	ATTACTCATAATTCTTGC	−355/−337	1
miR167c-5p	ATATATCATTCTTCTCTC	−142/−124	3
miR169b-5p	AGCTAGCTTACTTATTCC	−809/−791	5
miR171c-5p	ACCAAGCAAGCTTAT_GC	−966/−948	1
miR319c	GTAAAGCTTGGTTCTTTC	−363/−345	2
miR390a-3p	ATTAAGCTTCCTTCATTTC	−383/−364	2
miR394a	AACATGCATGGTGCATGC	−936/−918	1
miR396b-5p	TTCAAGCTT_CTTCTCTC	−631/−613	5
miR396b-3p	TTCAAGCTT_CTTCTCTC	−631/−613	5
miR401	ATTAGG_ATGCTTCTTGA	−914/−896	4
miR167a-3p	ATCTAACAT_CTTCTAGA	−504/−486	3
miR167a-5p	ATCTAACAT_CTTCTAGA	−504/−486	3
miR160c-3p	AATAAGGATTCTTCTTGT	−545/−558	5

## Data Availability

Data are contained within the article and Supplementary Materials.

## Supplementary Materials

The following supporting information can be downloaded at: https://www.mdpi.com/article/10.3390/plants15020227/s1 Identification of miRNAs responsive to a defined period of iron deficiency and resupply in *Arabidopsis thaliana*. Figure S1. Identification of potential metal-responsive cis-elements in promoters of *Arabidopsis thaliana* Fe-deficiency/recovery-responsive miRNAs. Motif analysis was performed within 2 kb promoter regions upstream of miRNA stem-loop structures. IDE1 motifs are denoted by purple squares; Figure S2. Principal component analysis (PCA) of *Arabidopsis thaliana* transcriptomes under Fe homeostasis perturbation; Figure S3. Predicted potential regulatory networks governing Fe deficiency/recovery-responsive miRNAs in *Arabidopsis thaliana* Integrated root and leaf miRNA-target networks; Table S1. The predicted IDE1 motifs were identified in the promoter regions upstream of the Fe deficiency-responsive genes *FIT1*, *FRO2*, and *IRT1* in *Arabidopsis*; Table S2. Computational prediction and functional annotation of target genes for IDE1-harboring miRNAs; Table S3. miRNA-mRNA binding characteristics of predicted targets for IDE1-associated miRNAs.

## Abbreviations

The following abbreviations are used in this manuscript:

FeD	Fe deficiency
Fol	Foliar spraying recovery
RE	Root exposure recovery
Fe	Iron
Mn	Manganese
Zn	Zinc
Cu	Copper
miRNA	MicroRNA
S	Sulfur
TCA	tricarboxylic acid
FRO2	Ferric reduction oxidase 2
IRT1	Iron-regulated transporter 1
FIT	Fe-deficiency induced transcription factor/basic helix-loop-helix protein 29
IDEs	Fe deficiency responsive elements
miRISCs	MicroRNA-induced silencing complexes
NF-YA	Nuclear factor Y A
TCP	Teosinte branched1/cycloidea/pcf
ARF	Auxin response factor
BRZ	Brassinazole
PAC	Paclobutrazol
PSII	Photosystem II
DEmiRs	Differentially expressed miRNAs
BLASTN	Homology-based algorithms
DEGs	Differentially expressed genes
HEMA1	Glutamyl-tRNA reductase family protein
LSU2	Response to low sulfur 2
NF-YA2	Nuclear transcription factor y subunit A2
TPR	Tetratricopeptide repeat
