# Supplementary material for: Identification of miRNAs Responsive to a Defined Period of Iron Deficiency and Resupply in Arabidopsis thaliana
Source: Plants (Basel). 2026 Jan 11;15(2):227. doi: 10.3390/plants15020227 (PMC12845292; doi:10.3390/plants15020227)
Supplement: Supplementary file 1 [file plants-15-00227-s001.zip › Supplementary figures.pdf]

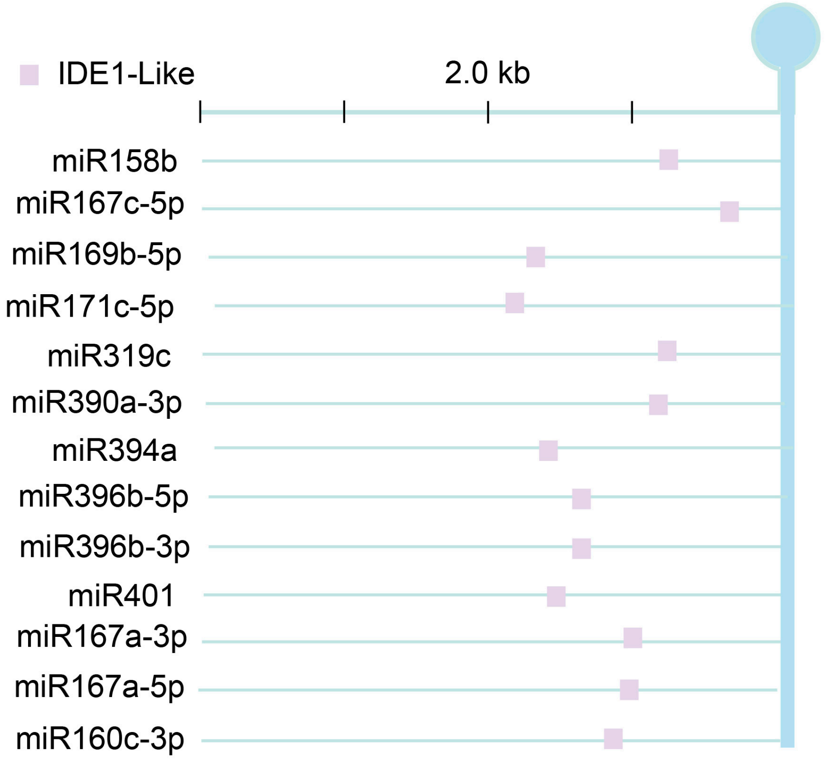

Supplementary Figure S1. Identification of potential metal-responsive cis-elements in promoters of *Arabidopsis thaliana* Fe-deficiency/recovery-responsive miRNAs. Motif analysis was performed within 2-kb promoter regions upstream of miRNA stem-loop structures. IDE1 motifs are denoted by purple squares.

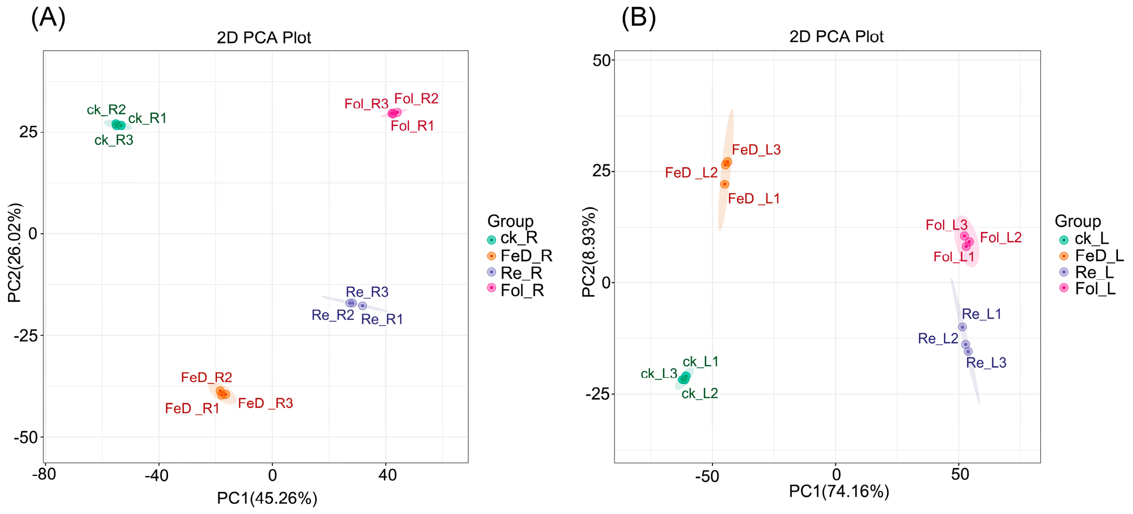

Supplementary Figure S2. Principal component analysis (PCA) of *Arabidopsis thaliana* transcriptomes under Fe homeostasis perturbation. (**A**), Root samples: Control (CK), Fe deficiency (FeD), resupply via medium (Re), and foliar resupply (Fol). (**B**), Leaf samples: Identical experimental regimes.

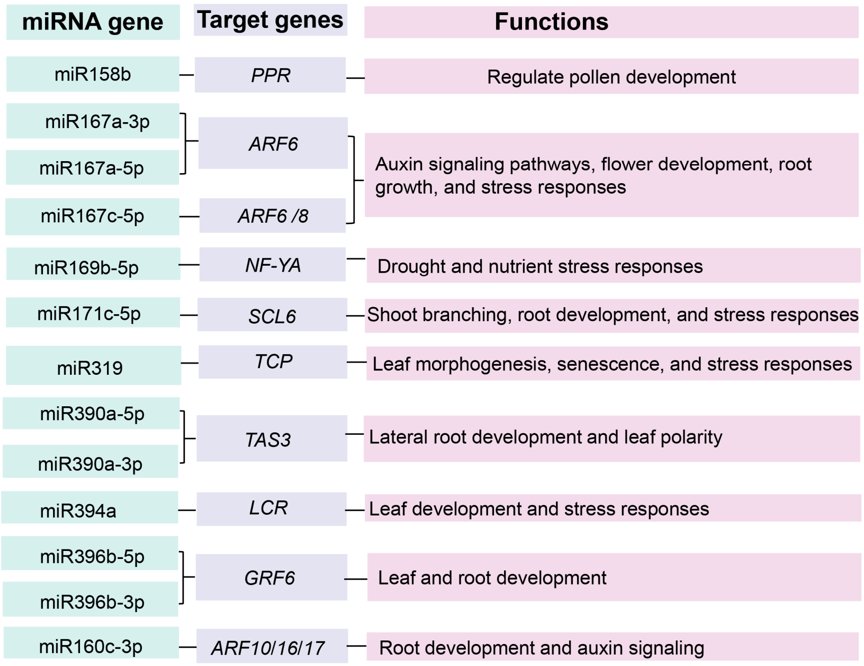

Supplementary Figure S3. Predicted potential regulatory networks governing Fe deficiency/recovery-responsive miRNAs in *Arabidopsis thaliana* Integrated root and leaf miRNA-target networks.
